# Study of Clinicoetiological Spectrum of Bicytopenia and Pancytopenia in Hospitalized Children

**DOI:** 10.7759/cureus.66255

**Published:** 2024-08-06

**Authors:** Gaurav Kumar, Sarita Verma, Sanjay Chavan, Aryan Gupta, Om Prasanth Reddy Avuthu, Shailaja Mane, Mridu Bahal, Balakrushna Garud, Shradha Salunkhe, Nakul Pathak

**Affiliations:** 1 Pediatrics, Dr. D. Y. Patil Medical College, Hospital and Research Centre, Dr. D. Y. Patil Vidyapeeth, Pune, IND

**Keywords:** leukemia, cytopenia, dengue fever/complications, infections, pancytopenia, bicytopenia

## Abstract

Background

The etiological profile of children with bicytopenia and pancytopenia has a very wide spectrum, ranging from transient causes like infections or nutritional deficiencies to bone marrow failure syndromes. Timely diagnosis and treatment impart favorable prognosis to this entity. There is a paucity of data regarding the etiology of cytopenia in hospitalized children at a tertiary center in India. Additionally, only a few studies have discussed the possible association between the severity of cytopenia at presentation and the possible etiology.

Methods

This is a cross-sectional observational study analyzing bicytopenia and pancytopenia in hospitalized children. Patient details, along with clinical findings and relevant investigations, were recorded on predesigned pro forma and analyzed statistically.

Results

Out of 202 children, 174 (86.13%) had bicytopenia, and 28 (13.86%) had pancytopenia, with a male predominance resulting in a male-to-female ratio of 1.65:1. The commonest age group affected was pre-adolescent age group (6-12 years). The causes of bicytopenia and pancytopenia in hospitalized children in the decreasing order of frequency were infections (65.84%), benign hematological disorders (18.81%), systemic illness (10.39%), and malignancies (4.95%). The cytopenia was more severe in children with pancytopenia than bicytopenia.

Conclusions

Infections outweigh the other causes of bicytopenia and pancytopenia. The severity of the cell line affected can help narrow down a diagnosis of cytopenia etiologies. Most of the children with bicytopenia and pancytopenia had treatable etiology and favorable outcomes.

## Introduction

Cytopenia refers to reduction in the number of blood cell components: red cells, white cells, or platelets. Bicytopenia occurs with a decrease in any two cell lines, while pancytopenia involves a reduction in all three cell lines. These conditions are common in hospitalized patients, stemming from various causes, ranging from transient infections to severe hematological disorders [1].

Bicytopenia and pancytopenia share overlapping causes like infections, nutritional deficiencies, drug-induced bone marrow suppression, and potentially life-threatening marrow failure or leukemia. Accurate diagnosis is crucial for guiding treatment and assessing prognosis. Peripheral smear analysis is essential, and bone marrow aspiration or biopsy is often needed for confirmation of diagnosis [1].

Cytopenia's etiology includes inadequate production (intrinsic marrow disorders), sequestration (e.g., hypersplenism), and increased destruction peripherally. Genetic factors like Fanconi anemia or acquired conditions, such as viral infections or autoimmune disorders, can contribute to cytopenia. Hypersplenism, autoimmune diseases like lupus, and conditions like paroxysmal nocturnal hemoglobinuria further complicate diagnosis and management [2].

Clinically, anemia leads to fatigue, breathlessness, and cardiac symptoms. Thrombocytopenia leads to bruising and mucosal bleeding, and leukopenia leads to increased susceptibility to infection [2].

This study aims to delineate the diverse causes of bicytopenia and pancytopenia in hospitalized pediatric patients, utilizing clinical and hematological parameters, including peripheral blood and bone marrow examinations.

## Materials and methods

Study design

We conducted this cross-sectional observational study at the Department of Pediatrics, Dr. D. Y. Patil Medical College, Hospital and Research Centre, Pune, over 22 months. The institutional ethics committee of Dr. D. Y. Patil Medical College, Hospital and Research Centre, Pune, approved the study (approval no. I.E.S.C./291/2022). A total of 202 children meeting the inclusion criteria aged six months to 14 years and admitted to the department during the study period (August 2022 to June 2024) who consented to participate were enrolled in the study. We included children aged 6 months to 14 years, with at least two of the following:

a.) Hemoglobin (Hb) <11 g/dL,

b.) Total leucocyte count (TLC) <4,000 cells/µL,

c.) Platelets <150,000/µL,

d.) Absolute neutrophil count (ANC) <1,500 cells/µL.

We excluded children aged less than six months or more than 14 years or those already diagnosed with bicytopenia or pancytopenia due to conditions like malignancy or aplastic anemia with repeated admissions or refusal of consent.

Data collection and statistical analysis

At their initial visits, detailed histories and clinical examination findings were recorded on a predesigned data collection form. We obtained laboratory data from the hospital’s informatics system and documented it in a Microsoft Excel spreadsheet (Microsoft Inc., Redmond, WA). Hemogram, peripheral smears, and red cell distribution width (RDW) were analyzed using the DxH 800 Hematology Analyzer (Beckman Coulter, Brea, CA) at the laboratory of Dr. D. Y. Patil Medical College, Hospital and Research Centre, Pune.

All the possible cytopenic permutations, which included anemia with neutropenia, anemia with thrombocytopenia, anemia with leukopenia, leukopenia with thrombocytopenia, neutropenia with thrombocytopenia, leukopenia with neutropenia, and pancytopenia were evaluated across all the etiologies to find out the possible association of specific cytopenic permutations in a specific etiology.

Statistical analysis included numbers, percentages, means, and standard deviations, with appropriate tests such as Fisher's exact test, analysis of variance (ANOVA), chi-square test, and p-values calculated using IBM SPSS Statistics for Windows, version 26.0.0.0 (IBM Corp., Armonk, NY). The chi-square or Fisher's exact test was used to compare the categorical variables. A p-value of <0.05 was considered statistically significant.

## Results

Out of 202 children, 174 (86.13%) had bicytopenia, and 28 (13.86%) had pancytopenia, with a male predominance observed in both groups. Overall, 126 (62%) were males, and 76 (38%) were females, resulting in a male-to-female ratio of 1.65:1, whereas that in bicytopenia and pancytopenia was 1.63:1 and 1.8:1, respectively (Table 1).

**Table 1:** Demographic profile of children with bicytopenia and pancytopenia

Demographic data	Bicytopenia (n = 174)	Pancytopenia (n = 28)
Male	108 (62.06%)	18 (64.28%)
Female	66 (37.93%)	10 (35.71%)
6 months-1 year	35 (20.1%)	2 (7.1%)
1-3 years	30 (17.2%)	6 (21.4%)
3-6 years	33 (19%)	2 (7.1%)
6-12 years	72 (41.3%)	16 (57.1%)
12-14 years	4 (2.3%)	2 (7.1%)

The median age of the entire study group was six years (range: 6 months-14 years), whereas that of children with bicytopenia and pancytopenia was five years (range: 6 months-13 years) and eight years (range: 7 months-14 years), respectively. The age group with the most children having bicytopenia and pancytopenia noted was the pre-adolescent age group (6-12 years), followed by the infant age group (6 months-1 year) in bicytopenia and the toddler age group in pancytopenia. The least number of cases of bicytopenia were noted in the adolescent age group (12-14 years), whereas nearly equal proportions of patients had pancytopenia in all the other age groups (Table 1).

The study divided all participants into two groups, bicytopenia and pancytopenia, comparing their respective signs and symptoms. Fever was the most prevalent symptom in both groups. Additionally, approximately half of the children in each group exhibited lethargy, vomiting, and cough. Notably, bleeding manifestations were significantly more frequent in children with pancytopenia (p < 0.05) (Table 2).

**Table 2:** Clinical profile of children with bicytopenia and pancytopenia Fisher's exact test is used here for the calculation of the p-value.

Presenting symptoms/signs	Bicytoepnia (n = 174)	Pancytopenia (n = 28)	p-value
Fever	163 (93.67%)	25 (89.28%)	0.39
Lethargy	89 (51.14%)	12 (42.85%)	0.41
Vomiting	74 (42.52%)	10 (35.71%)	0.49
Cough	65 (37.35%)	12 (42.85%)	0.57
Abdominal pain	45 (25.86%)	5 (17.85%)	0.36
Consanguinity	42 (24.13%)	8 (28.57%)	0.61
Skin changes	42 (24.13%)	4 (14.28%)	0.25
Breathlessness	33 (18.96%)	2 (7.14%)	0.12
Failure to thrive	28 (16.09%)	4 (14.28%)	0.24
Bleeding manifestation	17 (9.77%)	7 (25%)	<0.05
Joint pain	17 (9.77%)	3 (10.71%)	0.87
Short stature	12 (6.89%)	6 (21.42%)	<0.05
Jaundice	12 (6.89%)	5 (17.85%)	0.05
Weight loss	9 (5.17%)	4 (14.28%)	0.06
Abdominal distension	8 (4.59%)	2 (7.14%)	0.56
Pallor	104 (59.77%)	26 (92.85%)	<0.05
Hepatomegaly	51 (29.31%)	13 (46.42%)	0.07
Spleenomegaly	17 (9.77%)	8 (28.57%)	<0.05
Edema	12 (6.89%)	3 (10.71%)	0.47
Icterus	11 (6.32%)	6 (21.42%)	<0.05
Lymphadenopathy	10 (5.74%)	2 (7.14%)	0.77
Dysmorphism	5 (2.87%)	1 (3.57%)	0.84
Limb anomaly	1 (0.58%)	0 (0%)	0.68

On examination, the pancytopenia group had a statistically higher proportion of children with short stature, icterus, and splenomegaly compared to those with bicytopenia (Table 2). The difference in the proportion of all other signs and symptoms was not statistically significant.

Among bicytopenic patients, the most commonly affected cell line was platelets (87%), followed by red blood cells (60%) and white blood cells (52%).

Peripheral blood smear examination revealed thrombocytopenia as the most frequently noted abnormality (72%), followed by leukopenia (58%) and a microcytic hypochromic picture (57%). Circulating blasts were observed in a small percentage of both bicytopenia (3%) and pancytopenia (7%) cases.

**Figure 1:**
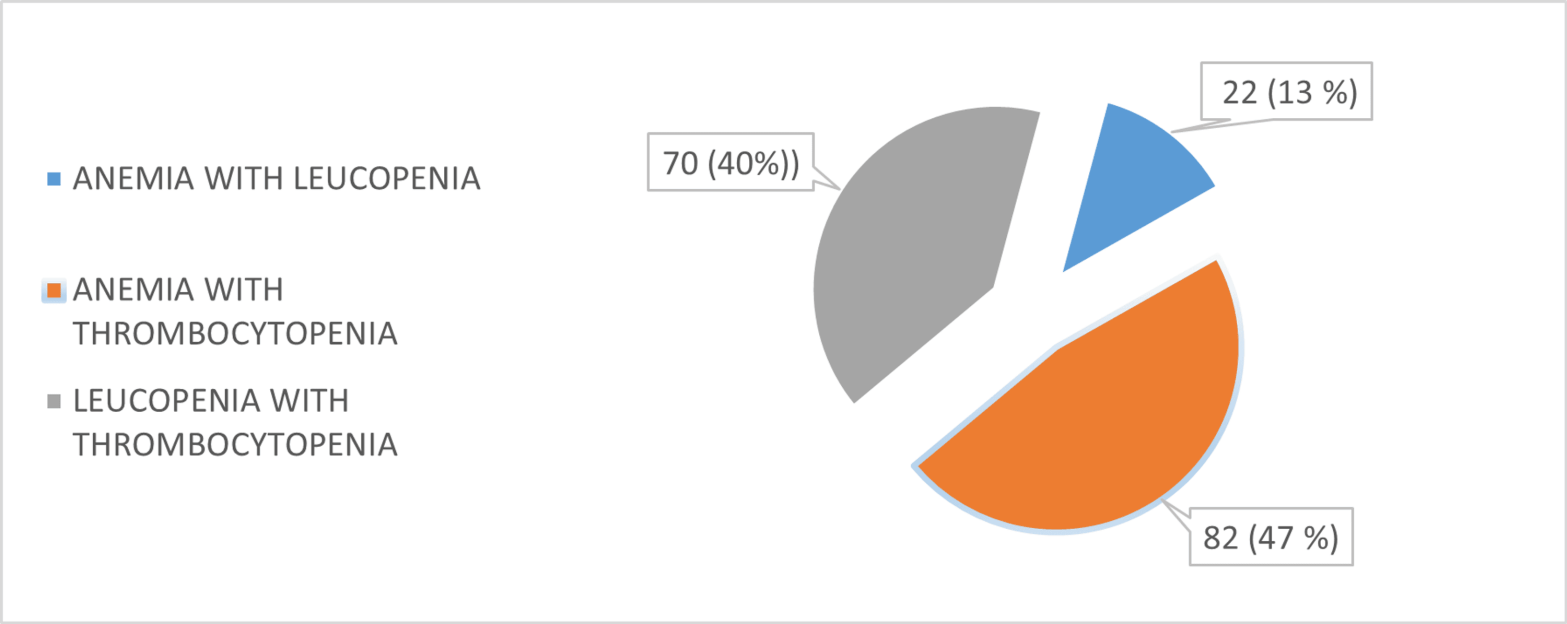
Spectrum of bicytopenia (pie chart)

Anemia with thrombocytopenia (41%) was the most common cytopenic profile in bicytopenia, followed by leukopenia with thrombocytopenia (35%) and anemia with leukopenia (11%) (Figure 1).

Comparative analysis between the bicytopenia and pancytopenia groups showed significantly lower mean levels of mean Hb, TLC, ANC, and platelets in the pancytopenia group, whereas mean corpuscular volume (MCV), mean corpuscular hemoglobin (MCH), and RDW were significantly lower in the bicytopenia group. MCHC levels did not differ significantly between the groups (Table 3).

**Table 3:** Comparison of mean CBC parameters in bicytopenia and pancytopenia ANC - absolute neutrophil count, CBC - complete blood count, MCH - mean corpuscular hemoglobin, MCHC - mean corpuscular hemoglobin concentration, MCV - mean corpuscular volume, RDW - red cell distribution width, TLC - total leucocyte count ANOVA test is used here for the calculation of the significance of the difference between two mean parameters and p-value.

Laboratory parameters	Bicytopenia	Pancytopenia	p-value
Mean hemoglobin	9.85	7.87	<0.05
Mean TLC	7,551.89	3,421.07	<0.05
Mean ANC	3,685.61	1,087.64	<0.05
Mean platelets	1.12	0.69	<0.05
MCV	73.76	79.45	<0.05
MCH	24.33	26.42	<0.05
MCHC	32.45	33.10	0.16
Mean RDW	17.80	20.65	<0.05

**Figure 2:**
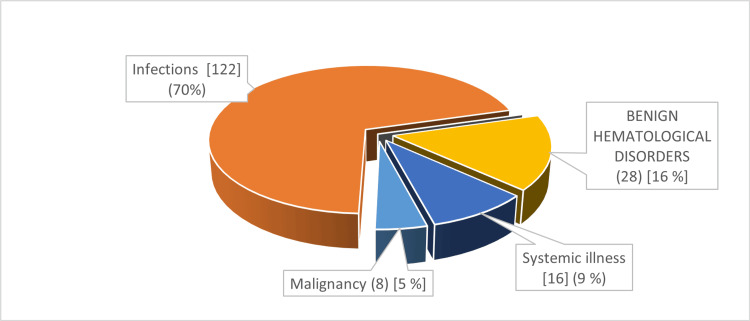
Etiological profile in bicytopenia (pie chart)

**Figure 3:**
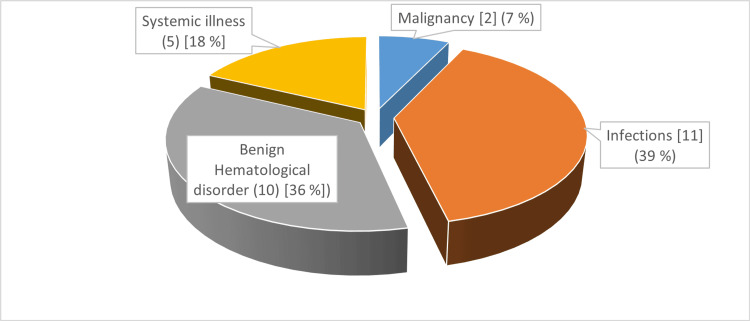
Etiological profile in pancytopenia (pie chart)

For the entire group with cytopenia, the etiologies noted were infections (65.74%), followed by benign hematological disorder (18.81%), systemic illnesses (10.39%), and malignancies (4.95%) (Table 4). A similar trend in frequencies was noted in both the bicytopenia and pancytopenia groups (Figure 2 and Figure 3).

**Table 4:** Etiologies of bicytopenia and pancytopenia in the total study group

Serial number	Diagnosis	Number (%) (n = 202)
1.	Infections	133 (65.84%)
a.	Dengue	87 (43.06%)
b.	Respiratory viruses	36 (17.82%)
c.	Sepsis	6 (2.97%)
d.	Enteric fever	3 (1.48%)
e.	Infectious mononucleosis	1 (0.49%)
2.	Benign hematological disorders	38 (18.81%)
a.	Dimorphic anemia	26 (12.87%)
b.	Hypoplastic anemia	3 (1.48%)
c.	Hemophagocytic lymphohistiocytosis (HLH)	4 (1.98%)
d.	Immune thrombocytopenic purpura (ITP) with iron deficiency anemia (IDA)	3 (1.48%)
e.	Lymphoproliferative disorder	1 (0.49%)
f.	Congenital hemolytic anemia	1 (0.49%)
3.	Systemic illness	21 (10.39%)
a.	Miscellaneous	17 (8.41%)
b.	Extrahepatic portal vein obstruction (EHPVO)	3 (1.48%)
c.	Hemolytic-uremic syndrome	1 (0.49%)
4.	Malignancy	10 (4.95%)
a.	Pre-B-acute lymphoblastic leukemia (ALL)	7 (3.46%)
b.	T-ALL	1 (0.49%)
c.	Neuroblastoma	1 (0.49%)
d.	Ovarian tumor	1 (0.49%)
1 + 2 + 3 + 4	Total	202

The study highlighted statistically significant variations in the presentation of different cytopenic permutations across etiological groups. The commonest cause of pancytopenia and leukopenia with thrombocytopenia was infection, while the most common cause of anemia with neutropenia noted was malignancy. For anemia with thrombocytopenia, benign hematological group and systemic illness were the common causes (Table 5).

**Table 5:** Spectrum of bicytopenia and pancytopenia in different etiological groups A - anemia, L - leukopenia, N - neutropenia, PAN – pancytopenia, T - thrombocytopenia The chi-square test is used in this table for the calculation of the significance of the difference between multiple proportions and the p-value.

Final diagnosis	A + L	A + T	L + T	A + N	L + N	N + T	PAN
Infections (133)	9 (7%)	43 (32%)	66 (50%)	8 (6%)	41 (31%)	41 (31%)	11 (8%)
Benign hematological disorders (38)	7 (18%)	22 (58%)	1 (3%)	5 (13%)	3 (8%)	2 (5%)	10 (26%)
Systemic conditions (21)	2 (10%)	12 (57%)	2 (10%)	0	0	0	7 (33%)
Malignancy (10)	3 (30%)	5 (50%)	0	5 (50%)	3 (30%)	2 (20%)	2 (20%)
p-value	<0.05	<0.05	<0.05	<0.05	<0.05	<0.05	<0.05

While comparing presenting symptoms and signs in different etiologies, fever was the most common presenting symptom in children with cytopenia, where almost all patients with malignancy were affected, followed by infections, whereas the fewest patients were affected with fever in systemic illnesses. Fever was statistically more significant in the malignancy group, followed by the infectious group and the benign hematological group (Table 6).

**Table 6:** Comparison of sex, consanguinity, signs, symptoms, and PICU admission in etiological groups PICU - pediatric intensive care unit, NA - not applicable The chi-square test is used here in this table for the calculation of the significance of the difference between multiple proportions and the p-value.

Parameters	Infections (n = 133)	Benign hematological (n = 38)	Systemic illness (n = 21)	Malignancy (n = 10)	p-value
Male	79 (58.95%)	23 (65.78%)	12 (57.14%)	6 (60%)	0.99
Consanguinity	28 (20.89%)	13 (34.21%)	7 (33.33%)	2 (20%)	0.29
PICU	27 (20.14%)	15 (39.47%)	9 (42.85%)	3 (30%)	<0.05
Fever	131 (97.76%)	33 (86.84%)	15 (71.42%)	10 (100%)	<0.05
Breathlessness	23 (17.16%)	9 (23.68%)	2 (9.52%)	0 (0%)	0.38
Cough	53 (39.55%)	16 (42.10%)	3 (14.28%)	3 (30%)	0.12
Abdominal pain	41 (30.59%)	3 (7.89%)	5 (23.80%)	2 (20%)	<0.05
Abdominal distension	1 (0.74%)	1 (2.63%)	4 (19.04%)	2 (20%)	<0.05
Vomiting	58 (43.28%)	14 (36.84%)	7 (33.33%)	3 (30%)	0.64
Bleeding manifestation	6 (4.47%)	9 (23.68%)	5 (23.80%)	3 (30%)	<0.05
Jaundice	4 (2.98%)	9 (23.68%)	3 (14.28%)	0 (0%)	<0.05
Lethargy	73 (54.47%)	20 (52.63%)	3 (14.28%)	4 (40%)	<0.05
Dysmorphism	5 (3.75%)	0 (0%)	1 (4.76%)	0 (0%)	0.82
Weight loss	4 (2.98%)	3 (7.89%)	3 (14.28%)	3 (30%)	<0.05
Joint pain	14 (10.44%)	0 (0%)	2 (9.52%)	4 (40%)	<0.05
Limb anomaly	1 (0.74%)	0 (0%)	0 (0%)	0 (0%)	NA
Skin changes	36 (26.86%)	9 (23.68%)	2 (9.52%)	1 (10%)	0.24
Failure to thrive	16 (11.94%)	10 (26.31%)	3 (14.28%)	1 (10%)	0.17
Short stature	7 (5.22%)	5 (13.15%)	5 (23.80%)	0 (0%)	<0.05
Lymphadenopathy	5 (3.73%)	0 (0%)	0 (0%)	7 (70%)	<0.05
Pallor	64 (47.76%)	38 (100%)	18 (85.71%)	10 (100%)	<0.05
Edema	6 (4.47%)	4 (10.52%)	4 (19.04%)	0 (0%)	<0.05
Icterus	5 (3.73%)	8 (21.05%)	3 (14.28%)	0 (0%)	<0.05
Hepatomegaly	38 (28.35%)	13 (34.21%)	5 (23.80%)	5 (50%)	0.43
Spleenomegaly	6 (4.47%)	11 (28.94%)	5 (23.80%)	2 (20%)	<0.05

Abdominal pain as a symptom occurred more frequently in children with infection followed by systemic illness, whereas abdominal distension as a presenting symptom was more common in children with systemic illness. A statistically significant proportion of patients presented with joint pain in patients with malignancy, followed by infection and systemic illness. Pallor and icterus were more common in children with benign hematological disorders (Table 6).

Splenomegaly was more common in benign hematological disorders and systemic illnesses than infection and malignancy. Short stature was more common in children with systemic illnesses, whereas lymphadenopathy was more common in malignancies. No statistical significance of other parameters was noted in various etiological groups. A significant proportion of patients with systemic illnesses and benign hematological disorders needed management in the critical care unit (Table 6).

There was a statistically significant difference in the mean Hb, WBC, ANC, and RDW, while mean platelets and MCV had no significant difference across various etiological groups. The lowest mean Hb, mean platelets, and widest RDW were found in benign hematological disorders, while the lowest mean TLC and MCV were noted in the infection group. The lowest mean ANC was noted in malignancy (Table 7).

**Table 7:** Mean CBC parameters in different etiological groups ANOVA test is used in this table for the calculation of the significance of the difference between multiple means and p-value.

Etiological groups	Mean Hb	Mean WBC	Mean ANC	Mean platelets	MCV	Mean RDW
Infections	10.74	5,883.95	2,858.05	1.09	74.05	16.72
Benign hematological disorders	6.55	9,367.89	4,386.44	0.94	75.87	23.21
Systemic illness	8.88	7,994.37	5,193.06	1.05	74.76	17.93
Malignancy	7.48	11,771	2,693	1.127	80.67	18.35
p-value	<0.05	<0.05	<0.05	0.53	0.30	<0.05

A statistically significant proportion of patients presented with bicytopenia than pancytopenia. There was no statistical difference in the mean Hb and mean RDW of children with bicytopenia and pancytopenia across all etiological groups. Whereas in children with infectious etiology, the mean ANC was significantly low in the pancytopenia group, whereas there was no statistically significant difference in the mean TLC, platelets, or MCV in the pancytopenia or bicytopenia groups with infectious causes (Table 8).

**Table 8:** Comparison of mean CBC parameters in bicytopenia and pancytopenia according to etiological groups ANC - absolute neutrophil count, CBC - complete blood count, MCH - mean corpuscular hemoglobin, MCHC - mean corpuscular hemoglobin concentration, MCV - mean corpuscular volume, RDW - red cell distribution width, TLC - total leucocyte count ANOVA test is used here in this table for the calculation of the p-value for the significance of the difference between two mean parameters, while Fischer's exact test is used to calculate the p-value for the significance of the difference between two proportions.

Etiological groups	Parameters	Bicytopenia (n = 174)	Pancytopenia (n = 28)	p-value
Infections (133)	Total number (%)	122 (91.72%)	11 (8.27%)	<0.05
	Mean Hb	10.83	9.67	0.07
	Mean TLC	5,944.91	3,385.45	0.11
	Mean ANC	2,896.32	820.18	0.05
	Mean platelets	1.11	0.84	0.21
	MCV	74.06	74.03	0.99
	Mean RDW	16.57	18.37	0.26
Benign hematological disorders (38)	Total number (%)	28 (73.68%)	10 (26.31%)	<0.05
	Mean Hb	6.88	5.6	0.13
	Mean TLC	11,483.57	3444	<0.05
	Mean ANC	5,590.1	1,016.2	<0.05
	Mean platelets	1.09	0.53	<0.05
	MCV	72.38	85.63	<0.05
	Mean RDW	22.38	25.53	0.36
Systemic conditions (21)	Total number (%)	16 (76.19%)	5 (23.80%)	<0.05
	Mean Hb	8.55	7.65	0.30
	Mean TLC	10,531.42	2,615.71	<0.05
	Mean ANC	7,096.57	1,597.14	<0.05
	Mean platelets	1.07	0.68	0.13
	MCV	73.93	76.41	0.58
	Mean RDW	18.85	16.1	0.25
Malignancy (10)	Total number (%)	8 (80%)	2 (20%)	<0.05
	Mean Hb	7.41	7.75	0.87
	Mean TLC	13,638.75	4,300	0.62
	Mean ANC	3,133.75	930	0.46
	Mean platelets	1.35	0.22	0.08
	MCV	77.86	91.9	<0.05
	Mean RDW	17.5	21.75	0.10

Children with benign hematological disorders had significantly low TLC, ANC, platelets, and MCV in the pancytopenia group (<0.05), while those with systematic conditions had significantly low TLC and ANC in the pancytopenia group with no significant difference in the mean platelet and MCV. Whereas children with malignancy had significantly low MCV in the bicytopenia group, with no statistical difference in the mean TLC, ANC, or platelet count in both groups (Table 8).

Nearly half of the children with pancytopenia 12 (42.85%) needed management in the intensive care unit, while only one-fourth of the bicytopenic 45 (25.66%) children needed management in the intensive care unit, which was statistically not significant. A nearly equal proportion of deaths (eight (4.59%) in bicytopenia and one (3.57%) in pancytopenia) were noted in both groups.

## Discussion

In our study cohort, 86% of patients had bicytopenia, and only 14% had pancytopenia. Male preponderance was observed in both bicytopenia and pancytopenia, similar to other studies. The majority of cases of bicytopenia as well as pancytopenia affected in our study were in the age group 6 to 12 years (Table 1). A possible explanation can be that infection was the most common etiology of cytopenia in our study, and among infections, dengue was the most common cause, which affects children with a mean age of nine years [3,4].

Fever was the most common symptom in our study, similar to other studies analyzing the etiology of bicytopenia (Table 9). As reported in other studies analyzing pancytopenia, our study also found significant bleeding manifestations in children with pancytopenia. Additionally, a significant proportion of our children presented with symptoms of upper respiratory tract infections, as viral respiratory infection also constituted a significant proportion of our study group (Table 4).

**Table 9:** Comparison of demographic, clinical, and etiological profile of the current study with other studies

Studies	Year	Sample size	Place	Mean age/common age group	Gender (M:F)	Signs/symptoms	Etiologies
Naseem et al. [1]	2011	571	Chandigarh	>5 years	2.9:1	1. Fever (57.96%) 2. Pallor (39.22%) 3. Hepatomegaly (54.64%) 4. Splenomegaly (45.88%)	1. Acute leukemia (55.34%) 2. Aplastic anemia (11.72%) 3. Megaloblastic anemia (6.58%) 4. Non-specific (13.58%)
Dosi et al. [11]	2018	107	Indore	2-16 years	1.5:1	1. Fever (42%) 2. Generalized weakness a. Bleeding b. Splenomegaly (41.12%) c. Hepatomegaly	1. Megaloblastic anemia (33.6%) 2. Acute leukemia (22.42%) 3. Mixed nutritional deficiency anemia (14%)
Sarbay et al. [6]	2019	130	Istanbul, Turkey	4.9 ± 4.86 years	1.69:1	1. Fever (43.8%) 2. Hepatosplenomegaly (22.3%) 3. Splenomegaly (6.2%)	1. Infections 2. Nutritional deficiency
Vijayakrishnan et al. [16]	2020	117	Kottayam, Kerala	9-12 years	0.9:1	1. Fever (67.52%) 2. Pallor (21.37%) 3. Hepatomegaly (28.21%) 4. Splenomegaly (21.37%)	1. Infections (47.86%) 2. ALL (22.22%)
Chouthai et al. [17]	2020	62	Ratnagiri, Maharashtra	10-12 years	1.44:1	1. Generalized weakness (82%) 2. Fever (81%) 3. Pallor (79%) 4. Hepatosplenomegaly (74%)	1. Megaloblastic anemia (40%) 2. Infections (26%) 3. Malignancies (23%)
Katoch et al. [18]	2021	50	Shimla	11-14 years	0.79:1	1. Fever (74%) 2. Generalized weakness (58%) 3. Pallor (86%) 4. Hepatomegaly (64%)	1. Infections (44%) 2. Leukemia (22%)
Current study	2024	202	Pune	6-12 years	1.65:1	1. Fever (93.06%) 2. Lethargy (50%) 3. Pallor (64.35%) b. Hepatomegaly (31.68%) c. Splenomegaly (12.37%).	1. Infections (65.84%) 2. Benign hematological disorder (18.81%) 3. Systemic illnesses (10.39%) 4. Malignancies (4.95%)

On examination, short stature was statistically significant in the children with pancytopenia. Additionally, jaundice was also more common in children with pancytopenia. The other clinical features were similar in frequency in both groups (Table 2).

The proportion of children presenting with hepatomegaly in children with bicytopenia and pancytopenia was comparable with other studies; however, our patients with bicytopenia had a lower proportion of splenomegaly (10%) as compared to other studies by Venkat and Bharani [5] showing 41% and Sarbay et al. showing 29% [6]. The possible explanation for this finding could be that the prevalence of splenomegaly in dengue is approximately 26% [3,4], whereas in respiratory viral infections, splenomegaly is negligible. These two infections constituted a major proportion of bicytopenia in this age group (Table 4).

In this study, infections were the major etiology of bicytopenia as well as pancytopenia (70.11% and 39.28%, respectively). Infections were mainly due to dengue, respiratory viruses, and sepsis. Hematological disorders were the second leading cause of bicytopenia (16%) and pancytopenia (25%). Hematological disorders mainly included dimorphic anemia and hemophagocytic lymphohistiocytosis (HLH). Systemic illnesses were the third major cause of bicytopenia as well as pancytopenia, 9% and 18%, respectively (Table 4) (Figure 2 and Figure 3).

In our study, the most frequent presenting symptoms in children with bicytopenia/pancytopenia were fever, cough pallor, lethargy, and vomiting (Table 2). Similar complaints have also been reported in similar studies (Table 9). These symptoms point toward a discrepancy in bone marrow cellularity that leads to a reduction in three of the cell lines. The low leukocytes make the patients prone to various infections causing fever and anemia, or low Hb, which translate into symptoms like pallor and lethargy. An early manifestation of neutropenia is often a sore throat, cough, chest, or soft tissue infection with a poor response to antibiotics. Hepatomegaly, splenomegaly, and lymphadenopathy were frequent clinical features that were assessed among patients in this study, which are comparable to several other studies reported from developing countries. Hypersplenism, characterized by splenomegaly and cytopenia(s), is due to peripheral pooling and destruction of cells in the enlarged spleen, resulting in pancytopenia.

We observed in this study that besides malignancies, aplastic anemia, and bone marrow failure syndromes that present as pancytopenia in pediatric patients, infections and megaloblastic anemia have also emerged as recognizable causes of varying degrees of cytopenia.

Dengue has been reported to be the most common infective cause of bicytopenia/pancytopenia in our population. In this study, of 133 patients (66%) with infections, dengue accounted for 87 patients (65.5%). Infection with flaviviruses, such as dengue virus, is associated with transient suppression of hemopoiesis. Thrombocytopenia (68.46%) is the most common clinical manifestation of dengue [7].

Tropical and subtropical areas experience dengue infection throughout the year, and the disease is closely correlated with the rainy season, temperature, vector fluctuation, and changing seasons. Most states in India report dengue outbreaks every year, making them a major cause of hospitalizations. Several factors contribute to the emergence of dengue fever, including ecological changes, climate change, epidemics of mosquito vectors, and changing demographics [8].

Respiratory virus infections are emerging causes of bicytopenia and pancytopenia, as reported by Fettah et al. [9]. A significant proportion of children with infectious etiology in our study had transient cytopenia following respiratory virus infection.

Sepsis is another infective cause in our study (4.5%) (Table 4). Sepsis causes pancytopenia through several mechanisms (marrow suppression, hypersplenism, and consumptive coagulopathy), which usually act in combination. Patients with pancytopenia may develop overwhelming sepsis without any focal sign of infection, with malaise and fever being the only clinical features [10].

In this study, three children were reported to have enteric fever. In enteric fever, pancytopenia is caused by varied mechanisms; bone marrow may undergo histiocytic hyperplasia along with hemophagocytosis or complete necrosis. Immune-mediated hemolysis, leukopenia, hypersplenism, and transient disseminated intravascular hemolysis are other contributory mechanisms. Varying degrees of cytopenia have been reported in many other series on enteric fever [11].

A statistically significant proportion of patients presented with bicytopenia than pancytopenia in all the etiological groups in this study (Table 8).

Hematological disorders were the second most common etiology of bicytopenia/pancytopenia in our study: 16% in bicytopenia and 25% in pancytopenia. The most common cause of benign hematological disorder in our study was dimorphic anemia secondary to nutritional causes. The most common nutritional cause of anemia is iron deficiency, although deficiencies in folate and vitamins B12 and A are also important causes [12].

Ineffective erythropoiesis, leucopoiesis, and thrombopoiesis due to increased programmed cell death in the absence of vitamin B12 or folic acid and decreased survival of precursors in peripheral blood are most commonly implicated in causing pancytopenia in megaloblastic anemia [11].

Neoplastic causes of pancytopenia in children cannot be overlooked. Even though neoplastic etiologies of pancytopenia in children are more common in developed countries, its prevalence in developing countries is also on the rise. In the present study, hematolymphoid malignancy was observed in 4% of cases. Pre-B-ALL constituted seven of the eight malignancies. A study by Krishnan et al. [13] from Kerala reported a statistically significant association between ALL and pancytopenia (p < 0.05). Acute lymphoblastic leukemia (ALL) is diagnosed in the US at a rate of approximately 2,500 cases per year, accounting for about one-third of all cases of childhood cancer. A timely diagnosis of ALL is very important, as the response to treatment is excellent in children. A statistically significant proportion of patients with malignancy presented with bicytopenia in our group. Though malignancies are known to present with pancytopenia more frequently [14], as in our cohort, the hematolymphoid malignancies had hyperleukocytosis, their ANC was more than 1,500, and they did not qualify as having pancytopenia as per definition.

The most common form of bicytopenia in our study was anemia with thrombocytopenia. As this study was conducted in a teaching institute, most of the patients belonged to the lower socioeconomic strata and had a high prevalence of nutritional anemia, and as dengue was the commonest etiology, most of the patients had concurrent thrombocytopenia leading to anemia with thrombocytopenia being the most common presentation among bicytopenia.

Similar findings were seen in other studies where Sharma et al. [15] and Vijayakrisnan et al. [16] reported that anemia with thrombocytopenia was the most common form of bicytopenia.

Additional analysis of our study revealed that a significant proportion of patients with malignancy had anemia with leukopenia as compared to the other etiological groups, whereas a significant proportion of anemia with thrombocytopenia was more common in benign hematological disorders and systemic conditions, the proportion of leukopenia with thrombocytopenia was significant in the infectious etiology. Anemia with neutropenia occurred more significantly in the malignancy group. Leukopenia with neutropenia constituted a significant proportion of the infectious etiology, whereas neutropenia with thrombocytopenia was also more prevalent in the infectious etiology (Table 5).

The clinical profile and etiologies of bicytopenia/pancytopenia, along with age and sex preponderance reported by researchers in various studies the world over, are summarized in Table 9.

The mean Hb of the total study cohort in our study was 9.58 g/dL, which was comparable to the study conducted by Sarbay et al. [6] (Table 10). Studies by Venkat and Bharani [5] and Agarwal et al. [17] have reported a lower mean Hb in comparison to our study. This could be because the two studies had a significant proportion of cases of malaria, which is known to cause anemia and nutritional anemias. Whereas, in our study, only 65% of the patients had anemia, and dengue was the commonest etiology of the study group, which is known to present with hemoconcentration and falsely elevated Hb.

**Table 10:** Comparison of hematological parameters of our study with various studies ANC - absolute neutrophil count, Hb - hemoglobin, MCV - mean corpuscular volume, WBC - white blood cell

Studies	Entity	Mean parameters
Sarbay et al. [6]	Bicytopenia/pancytopenia	Mean Hb: 9.3 ± 2.3; mean WBC: 10.207 ± 39.781; mean ANC: 1,515 ± 1,418; MCV: 80 ± 13.1; mean platelet: 118.823 ± 93.645
Venkat and Bharani [5], Vadodara, India	Bicytopenia/pancytopenia	Mean Hb: 6.90 ± 2.49; mean WBC: 11,425 ± 34,115.95; mean platelet: 72,543.85 ± 93,781.68
Agarwal et al. [19], Uttar Pradesh, India	Bicytopenia/pancytopenia	Mean Hb: 6.335; mean WBC: 21,625; MCV: 92.32; mean platelet: 58,835
Current study	Bicytopenia/pancytopenia	Mean Hb: 9.58 ± 2.69; mean WBC, 6,979.30 ± 7,740.30; mean ANC: 3,325.5 ± 4,151.04; MCV: 74.55 ± 11.25; mean platelet: 106,000 ± 67,000

The mean WBC of the total study cohort was 6,979 cells/µL in our study, which was lower than other studies conducted because, in our study, dengue was the commonest etiology of the study group, which presents with decreased TLC (Table 10).

The median ANC of the total study cohort was 3,800 cells/µL in our study, which was lower than other studies conducted because, in our study, dengue was the commonest etiology of the study group, which presents with decreased TLC and decreased ANC (Table 10).

The median platelet of the total study cohort was 106,000/µL in our study, which was higher than in other similar studies. This was because we had enrolled the children with bicytopenia/pancytopenia at the initial presentation, where most of the patients with infectious etiologies had borderline low platelets, which later decreased in the course of the disease (Table 10).

Table 11 shows the comparison of hematological profiles in etiological groups in various studies. In this study, the etiological group with the lowest mean Hb was aplastic anemia, followed by malignancies and infections, which is comparable with the results reported by Sharma et al. [15].

**Table 11:** Comparison of hematological parameters across etiological groups in various studies and our study ANC - absolute neutrophil count, Hb - hemoglobin, WBC - white blood cell

Studies	Entity	Infections	Megaloblastic anemia	Aplastic anemia	Leukemia
Sharma et al. [15]	Bicytopenia/pancytopenia	Mean Hb: 8.015 ± 2.3; mean WBC: 5,722.05 ± 7200; mean platelets: 52,000 ± 3,0120	Mean Hb: 5.32 ± 1.84; mean WBC: 3,093.32 ± 1,723; mean platelets: 120,000 ± 135,100	Mean Hb: 4.8 ± 2.5; mean WBC: 1,495 ± 802.55; mean platelets: 12,500 ± 8,185	Mean Hb: 5.75 ± 2.6; mean WBC: 14,600 ± 16,180; mean platelets: 42,200 ± 38,700
Bhatnagar et al. [20]	Pancytopenia	Mean Hb: 6.75; mean ANC: 787.5; mean platelets: 58,625	Mean Hb: 6.3; mean ANC: 650; mean platelets: 44,931	Mean Hb: 6.6; mean ANC: 450; mean platelets: 12,500	Mean Hb: 6.9; mean ANC: 372; mean platelets: 19,000
Current study	Bicytopenia/pancytopenia	Mean Hb: 10.74; mean WBC: 5,833.95; mean ANC: 2,858.05; mean platelets: 109,000	Mean Hb: 6.75; mean WBC: 9,710.85; mean ANC: 4,501.31; mean platelets: 102,000	Mean Hb: 4.16; mean WBC: 2,133.33; mean ANC: 589; mean platelets: 15,600	Mean Hb: 7.48; mean WBC: 11,771; mean ANC: 2,693; mean platelets: 112,700

In this study, the lowest mean TLC was observed in the group of infections. The malignancy group presented with hyperleukocytosis, whereas cases of benign hematological disorder and systemic illness do not significantly affect TLC. The mean TLC observed in our study was comparable with that of the study conducted by Sharma et al. [15].

In the present study, the lowest mean platelets were noted in the aplastic anemia group, which is comparable with the study conducted by Sharma et al. [15].

In this study, there was no significant difference in the hematological profile between the bicytopenic and pancytopenic groups having infections (Table 8), while in benign hematological disorders, the values for mean TLC, ANC, and platelets were significantly lower in the pancytopenic group as compared to the bicytopenic group, and the MCV values were significantly higher in the pancytopenic group. In cases with systemic conditions, the mean TLC and ANC values were significantly lower in the pancytopenic group as compared to the bicytopenic group. In cases of malignancy, the MCV values were significantly higher in the pancytopenic group.

This observational study also aimed to study factors associated with the outcome in bicytopenic and pancytopenic patients. The patient's outcome depends on the etiology of pancytopenia, treatment modalities, supportive care, and the era in which results are analyzed. Advances in the management of diseases lead to better recovery rates over time. The majority of the cases that presented with bicytopenia and pancytopenia had treatable etiologies and favorable outcomes. A nearly equal proportion of deaths (eight (4.59%) in bicytopenia and one (3.57%) in pancytopenia) were noted in both groups, mainly due to multiorgan failure secondary to sepsis and severe HLH.

Our study had several important limitations, such as the non-inclusion of socioeconomic and cultural parameters, which greatly affected the etiologies of cytopenia. This study included patients from a single center; a multicentric study from different hospitals would have enabled us to describe the prevalence of these etiologies in a precise manner. Another limitation of this study was that it was conducted over a limited period of time, which is not sufficient to predict the outcome in children with acute leukemia and other chronic disorders. Investigations like viral antigen and antibody testing and genetic analysis for storage disorders were not done due to the economic constraints of the patients, so in a few cases, an etiological diagnosis could not be made. We empirically assigned diagnoses based on signs and symptoms and treated them.

## Conclusions

Bicytopenia/pancytopenia in pediatric patients has a diverse range of causes, including nutritional deficiencies, infections, autoimmune disorders, and neoplastic conditions. Bicytopenia is more prevalent than pancytopenia and shows varied etiologies, with males slightly more affected. Common symptoms include fever and lethargy. Infections, particularly viral infections like dengue and respiratory viruses, are the leading causes of both bicytopenia and pancytopenia, followed by benign hematological disorders. ALL is the most common malignant cause of pancytopenia. Children presenting with fever, leukopenia, and thrombocytopenia should be evaluated for infectious etiologies, including dengue fever, respiratory infections, enteric fever, and septicemia. Anemia with thrombocytopenia or neutropenia in febrile children warrants consideration of acute leukemia, necessitating a peripheral blood smear and bone marrow examination for diagnosis. Improving immunization programs and initiating empirical antibiotic therapy can help prevent infectious causes of pancytopenia. Addressing nutritional deficiencies through dietary improvements and vitamin supplementation is crucial. Pancytopenia tends to manifest more severe symptoms compared to bicytopenia. Most cases of bicytopenia/pancytopenia in this study had favorable outcomes, but unexplained cases should be closely monitored for potential malignancy development in the future.
